# Stigma of Mental Illness in Germans and Turkish Immigrants in Germany: The Effect of Causal Beliefs

**DOI:** 10.3389/fpsyt.2019.00046

**Published:** 2019-02-13

**Authors:** Ulrike Von Lersner, Julia Gerb, Serdar Hizli, Daniel Waldhuber, Anton Felix Wallerand, Malek Bajbouj, Georg Schomerus, Matthias C. Angermeyer, Eric Hahn

**Affiliations:** ^1^Department of Clinical Psychology, Humboldt University of Berlin, Berlin, Germany; ^2^Friedrich von Bodelschwingh-Klinik, Berlin, Germany; ^3^Charité Medical University of Berlin, Berlin, Germany; ^4^Department of Psychiatry and Psychotherapy, University of Greifswald, Greifswald, Germany; ^5^Center for Public Mental Health, Gösing am Wagram, Austria

**Keywords:** stigma, mental illness, causal beliefs, social distance, cross-cultural psychology

## Abstract

**Background:** Stigma poses an additional burden for people suffering from mental illness, one that often impairs their social participation and can prevent them from seeking adequate help. It is therefore crucial to understand how stigma develops in order to counteract it by setting up effective evidence-based anti-stigma interventions. The present study examines the effect of causal beliefs on stigmatizing behavioral intentions, namely people's desire to distance themselves from persons with mental illness. In addition, we draw cross-cultural comparisons between native Germans and Turkish immigrants to investigate the influence of culture on stigma and causal beliefs and to broaden knowledge on the biggest immigrant group in Germany and on immigrants in Western countries in general.

**Methods:** *n* = 302 native Germans and *n* = 173 Turkish immigrants were presented either a depression or a schizophrenia vignette. Then, causal beliefs, emotional reaction and desire for social distance were assessed with questionnaires. Path analyses were carried out to investigate the influence of causal beliefs on the desire for social distance and their mediation by emotional reactions for Germans and Turkish immigrants, respectively.

**Results:** We found an influence of causal beliefs on the desire for social distance. Emotional reactions partly mediated this relationship. Causal attribution patterns as well as the relationship between causal attributions and stigma varied across both subsamples and mental illnesses. In the German subsample, the ascription of unfavorable personal traits resulted in more stigma. In the Turkish immigrant subsample, supernatural causal beliefs increased stigma while attribution to current stress reduced stigma.

**Conclusion:** Our study has implications for future anti-stigma interventions that intend to reduce stigmatization of mentally ill people. Targeting the ascription of unfavorable personal traits and supernatural causal attributions as well as promoting current stress as the cause for mental illness appears to be of particular importance. Also, the mediating influence of emotional responses to causal beliefs needs to be addressed. Furthermore, differential interventions across cultural groups and specific mental illnesses may be appropriate.

## Introduction

People with mental disorders often are subject to stigmatization: they are feared, avoided, patronized or, more subtle, incapacitated by exaggerated benevolence (1, 2). In addition to the adversities of having a mental illness as such, those affected are often further burdened by the negative social impact of the stigma associated with it. Thereby, the impact of public stigma does not stop at facing negative attitudes: people with mental illness are e.g., less likely to be hired (3, 4), thereby being put at an economic disadvantage. At the same time they are more likely to be falsely accused of a violent crime (5), and to become victims of harassment in their communities (6). Moreover, and perhaps even more bleak, stigma prevents people from seeking help of mental health services (7), leading to further preservation of impaired health and social disadvantage. This is further aggravated by the fact that, opposed to public perception (8), fear and stigma of the mentally ill seems to have remained unchanged or even increased over the past decades (9, 10). It is, therefore, of vital importance and apparent urgency to seek a better understanding of the pathways leading to the stigma associated with mental illness to effectively counteract its damaging consequences.

Research on stigma of mental illness has gained momentum since the 1960s, with Goffman's (11) pioneering work. In the past decades, stigma has been defined in various ways. An influential definition was introduced by Link and Phelan (12) according to whom stigma occurs as the convergence of four interrelated components: (1) people recognize and label human differences, (2) people link those differences with undesired characteristics, (3) people perform a certain degree of separation of “us” from “them,” (4) the labeled persons suffer status loss and discrimination. Corrigan and Watson (13) propose a similar model in which they map three sequential components of stigma: stereotypes, prejudices (that represent the emotional and cognitive response to stereotypes) and discrimination (which is the behavioral reaction that follows prejudices). Discrimination is, therefore, an outcome of the stigma process, whereby the two terms are sometimes used interchangeably. Discrimination can take several forms; example, Link and Phelan (12) distinguish between individual and structural discrimination as well as self-stigmatization. Individual stigma is often assessed by inquiring the *desire for social distance* (14–17) that is, how much distance people wish to put between themselves and the other person in certain social situations (18). More specifically, in respect to mental illness, it represents the readiness to accept mentally ill people as friends, neighbors, co-workers or to spend time socializing with them (17, 19). The desire for social distance is, therefore, a measure of personal rejection since it reflects upon how a specific person A discriminates a person B (12). The desire for social distance thus poses the opposite of social acceptance. The present study is based on the assumption that the expressed desire for social distance is a valid indicator of discriminatory behavior based on stigma or serves as a proxy for it (15).

The concept of how the general public explains mental illness is usually referred to as *causal beliefs* and is understood as one aspect of the explanatory models of mental illness. They further encompass ideas about its definition, severity and prognosis of an illness as well as treatment preferences (20). Common causal beliefs held in Western societies are the attribution of mental illness to stress or biological factors such as brain disease and genetics (21, 22). Moreover, intrapersonal causal beliefs and the ascription of unfavorable personal traits, such as being of weak character, are also thought to play a role (16, 21), whereas mental illness is only rarely attributed to supernatural causes, such as receiving a punishment from God or being possessed (21). When causal attributions for different mental disorders are compared inconsistent findings are reported. For example, depression is often found to be attributed to more to current stress than schizophrenia, while schizophrenia is more often attributed to biogenetic or supernatural causes than depression (16, 23, 24).

Various studies link causal beliefs to varying degrees of stigmatizing attitudes and behaviors in the social environment of those affected. Some studies find causal attribution to current stress to be associated with less desire for social distance (19, 25) while in other studies no association was observed (26, 27). A similar pattern of results appears for chronic stress such as childhood adversities: while it is positively associated with social acceptance in some instances (28) no associations (27) or mixed results were reported in other studies (16, 29).

Particular attention has been given to the effect of biological and genetic (from now on referred to as “biogenetic”) causal beliefs, due to the influence if biological perspectives in clinical research, and its parallel increase in prevalence among the general population of several Western countries over the last decades (10, 30). Biogenetic causal beliefs have long been assumed to reduce stigma and have therefore been used in anti-stigma intervention initiatives pushing the notion of mental illness as “an illness as any other” [for example see (31)]. However, endorsement of biogenetic causal beliefs was not accompanied by a corresponding decrease in stigma (10, 32). Meanwhile a substantial amount of research accumulated evidence for the opposite, suggesting that biogenetic causal beliefs may strengthen some aspect of stigma as a perception of more pessimism and dangerousness (16, 33). A meta-analysis by Kvaale et al. (34) also found that biogenetic explanations seem to reduce blame but induce pessimism. Furthermore, intrapersonal as well as supernatural causal beliefs also seem to increase people's desire for social distance (16, 19).

According to Schomerus et al. (29), causal beliefs can affect social acceptance through different mechanisms: by allocation of responsibility, by the attribution of divergence and dangerousness, and by implicating a perception of prognosis. In a study examining the relationship between causal attributions and desire for social distance in a representative sample in Germany, Schomerus et al. (29) reported biogenetic causal beliefs to be associated with a greater desire for social distance for both schizophrenia and depression. Interestingly, this result was reported for schizophrenia, but not for depression in the meta-analysis by Kvaale et al. (34). In Schomerus et al. (29) these effects were mediated by an increase in perceived dangerousness and differentness as well as decreased onset responsibility, implying that there was no direct effect of biogenetic beliefs. Conversely, attribution to current stress resulted in higher social acceptance in schizophrenia, whereas no significant effect could be observed for depression. In summary, Schomerus' results demonstrate the substantial influence of mediator variables as well as notable differences across the examined mental disorders.

A subsequent study by Angermeyer et al. (23) examined the influence of biogenetic causal beliefs on social acceptance and additionally directed their attention to how this influence is mediated by the participant's emotional reactions. The assumption of mediation by emotional reactions is derived from the stigma concept of Corrigan and Watson (13). Angermeyer et al. (23) reported causal attribution to brain disease to be associated with a greater desire for social distance directly as well as indirectly through the mediation of fear. This effect was present for schizophrenia vignettes, but not in depression. Expectedly, fear was positively, and prosocial emotions were negatively associated with desire for social distance.

Both studies are focusing on a small set of causal beliefs; hence supernatural and intrapersonal causal beliefs were not considered. In the present study, we aim toward the inclusion of a broader spectrum of causal beliefs. In line with Angermeyer et al. (23), we also assumed that the emotional reaction of the participants will mediate the effect of causal beliefs on stigma.

Research of stigma and the perception of mental illness, in general, has mostly focused on Western populations (10, 35, 36). The available research on this topic shows with high consistency: mentally ill people all around the world are subjected to some form of discrimination (36, 37). Stigma of mental illness, therefore, seems to be a rather universal phenomenon (38–40). However, the particular experience of stigmatization, its meaning, practices and results vary across cultures (40–44). Furthermore, causal beliefs to appear to be influenced by cultural factors, differing across countries (10, 16, 45, 46) as well as across ethnic groups within countries (47–49). Turkish immigrants and their descendants pose the biggest minority group in Germany, with a number of 2.8 million people accounting for approximately 3.4% of the German population (50)^1^. By the assessment of a subsample of Turkish immigrants additionally to a subsample of native Germans the present study intends to counteract the under-representation of research on immigrants in Western countries. In the simplistic dichotomy between traditional and modern medicine, Turkish immigrants are usually being allocated to the traditional side (51). It is therefore of interest if cross-cultural differences can be observed despite the named similar exposure and social environment.

An investigation of public attitudes toward schizophrenia in rural Turkey suggests a high attribution to social problems (75% agreement) and weak personality (61.5% agreement) as a cause for the mental illness (52). Concerning these categories of causal beliefs, the findings are in line with those of Angermeyer's study with Germans (21). However, Taskin et al. (52) did not assess the endorsement of supernatural causal beliefs, which appear more prevalent among non-Westerners (48, 49, 53) and biogenetic causal beliefs, which have been of particular scientific interest in the recent past. Another study that investigated Turkish nationals in mainly urban areas with a broader set of potential causal attributions likewise found social causes to be the most common causal attributions for the assessed mental illnesses, namely depression and schizophrenia (54). Furthermore, attribution to social causes was more prevalent for depression while attribution to biogenetic causes was more prevalent for schizophrenia. In sum, Utz (54) showed a very similar attribution pattern as found in Western samples, however, a large proportion of the sample consisted of young highly educated participants from Istanbul.

A study conducted among mentally ill persons of Turkish origin in Germany reported a higher prevalence of supernatural beliefs in Turkish immigrants compared to German patients, but also more agreement on social causes and intrapersonal causes (47). Germans, in turn, attributed mental illness more frequently to smoking, alcohol and conflicts in the family of origin (47). A semi-qualitative study by Vardar et al. (51) took an exploratory approach using the method of free listing and reports that supernatural causal beliefs were not among the most frequently named neither by Germans nor by Turkish immigrants. Furthermore, Vardar et al. (51) found genetic causes to be more frequently named by Germans than by Turkish immigrants. To our best knowledge, hitherto no research has been conducted on the relationship between stigma and causal beliefs among Turkish immigrants in Germany.

Therefore, the aim of the study is two-fold. On the one hand, its objective is to investigate how stigma of mental illness is influenced by causal beliefs and how this influence is mediated by the emotional reaction. Building on the work of Schomerus et al. (29) and Angermeyer et al. (23) we intend to inspect a wider set of causal beliefs also taking into account intrapersonal and supernatural causes. On the other hand, we strive to address the lack of data on non-Western immigrants in Western countries in general and on Turkish immigrants in Germany in particular. We examine whether there are cross-cultural differences in causal beliefs, stigma and emotional reactions. The two aims will be pursued by utilizing an implementation of a path analyses in both subsamples.

Based on previous work we expect biogenetic causal attributions to be associated with a higher desire for social distance. Intrapersonal and supernatural causal beliefs are likewise predicted to be linked to more desire for social distance. Emotional reactions are assumed to mediate the influence of causal beliefs on the desire for social distance, whereby negative emotions, such as fear and anger, are expected to be associated with more desire for social distance. Positive prosocial emotions, in turn, are expected to be associated with less desire for social distance. Participants are expected to experience more negative emotions (55) and express more desire for social distance toward a person suffering from schizophrenia compared to depression. Regarding group differences, we assume Turkish immigrants to more frequently attribute mental illness to supernatural and intrapersonal causes, whereas Germans are expected to indorse biogenetic causes more frequently. No specific hypotheses are made about differences in the extent of social distance desired by the two subsamples as well as the nature of differences in its relationship with causal beliefs, thereby taking an exploratory approach in this area.

## Method

### Procedure

Participants, native Germans as well as Turkish immigrants, were approached on the street, in citizens registration offices and town halls in different districts of the city of Berlin between March and October 2017. The questionnaire took approximately 20 min to complete. All subjects received written information about study duration as well as the institutions involved and signed an informed consent before participation. The consent further emphasized that participation is voluntary and could be canceled at any time. Participants did not receive financial compensation. The aim of the study was described as the assessment of people's opinion on “certain problems” that would be presented in a short story. This was done to prevent confounding the subsequent answers by using terms related to clinical psychology/psychiatry. All survey procedures were approved by the Ethics Committee for Psychological Research at the Humboldt University Berlin and were conducted in accordance with the Helsinki Declaration.

Since it became evident the response rate of Turkish immigrants would be low, a problem that has already been reported in other studies (56), we adapted our data acquisition technique according to a qualitative study by Dingoyan et al. (57) in order to enhance participation rates. Thus, we established cooperation with numerous Turkish associations as well as eminent figures of the Turkish community in Berlin and asked them for distribution of the questionnaires among their members, colleagues, friends and acquaintances.

### Sample

In total, 302 Germans and 173 Turkish immigrants completed the questionnaires, leading to a total of *N* = 475 subjects. Table 1 presents socio-demographic data on the two samples. Germans and Turkish immigrants did not differ in gender, age and civil status, whereas significant differences were found in education, occupation, income and religion.

**Table 1:** Sociodemographic data of the two subsamples.

	**Nativegermans**	**Turkishimmigrants**	**Statistics**
	***n* = 302**	***n* = 173**	
**GENDER %**			
Male	43.71	39.31	*χ2*_(2)_ = 2.784 *p* = 0.249
Female	55.29	60.69	
Other	0.99	0.00	
**Age** *M* (*SD*)	39.08	38.63	*W* = 25,866 *p* = 0.858
	(15.49)	(12.83)	
**EDUCATION %**			
Advanced (Abitur)^a^	85.10	61.85	*χ2*_(2)_ = 31.916 *p* < 0.001^***^
Secondary school	14.90	38.15	
**OCCUPATION %**			
Employed	54.64	50.87	*χ2*_(5)_ = 14.901 *p* = 0.011^*^
University student	17.88	17.92	
Unemployed	5.63	8.09	
Retired	8.61	4.05	
Homemaker	1.32	6.94	
Other	11.92	12.14	
**CIVIL STATUS %**			
Single	58.61	56.65	*χ2*_(2)_ = 0.102 *p* = 0.749
Married/in a partnership	41.39	43.35	
**Income in**€	1308.99	894.14	*W* = 34,751 *p* < 0.001^***^
*M* (*SD*)	(1068.49)	(586.02)	
**RELIGION** ***%***			
Muslim	0.66	69.36	*χ2*_(3)_ = 295.41 *p* < 0.001^***^
Christianity	35.43	1.73	
None	61.92	23.12	
Other	1.99	5.78	

* *p < 0.05*,

*** *p < 0.001*.

a *Abitur is the highest school leaving qualification in Germany. It corresponds to the English Advanced Level and is required for university entrance*.

In the Turkish immigrant subsample, 59.5% of participants were born in Turkey and immigrated to Germany at some point in their life, while the other 40.5% were born and grew up in Germany. Those participants who immigrated themselves relocated to Germany between 1965 and 2017 (*Mdn* = year 1989) being of an average age of 18.9 years (*SD* = 10.38). Nearly half of the Turkish immigrant participants were Turkish citizens (49.1%), 38.2% held a German citizenship and 10.4% were in possession of both, the German and Turkish, citizenship.

### Measures

To adjust for proficiency in German language in the Turkish subsample, subjects with a Turkish background could choose their preferred language of assessment, German or Turkish. For this purpose, the questionnaire was translated into Turkish using a collaborative and iterative method (58). A native Turkish speaker translated the questionnaires from German into Turkish. Three independent native speakers verified the Turkish translation for comprehension and compared it to the German version for correctness.

#### Case Vignettes

The survey began with the presentation of an unlabeled ungendered case vignette describing a psychiatric case history. The case vignettes described symptoms of either depression or schizophrenia according to criteria of the Diagnostic and Statistical Manual of Mental Disorders, (DSM-IV) and were validated in numerous previous studies (21, 29, 59, 60). Unlabeled vignettes are a well-established method in the research of attitudes toward people with mental illness. They offer valuable advantages, such as not using a diagnostic label and therefore avoiding possible prejudices associated with it, and the possibility of portraying the clinical picture of the respective mental disorder in its manifold aspects. Further, using case vignettes allows for standardization of the base of assessment ensuring that every participant is evaluating the same matter at hand. The two vignettes used in the present study were assigned randomly.

#### Causal Attributions

Participants were presented a checklist of 17 possible causal attributions and asked to indicate their agreement to the respective item being a cause of the clinical symptoms depicted in the vignette. The 5-point Likert scale ranged from 1 = “is definitely a cause” to 5 = “is definitely not a cause.” The checklist consisted of items used in previous studies in this field (21, 29). Adaptations were made on the basis of a qualitative free listing study performed on Turkish immigrants in Berlin (51, 54). Each item was referring to one of five causal belief categories: biogenetic causes, childhood adversities, current stress, supernatural causes and intrapersonal causes (21, 29, 54).

A confirmatory factor analysis (CFA) performed to verify the theorized categories in both groups. A subsequent analysis revealed the misfit in both groups to stem from an item pair with highly similar phrasing (“strains and worries in the partnership” and “strains and worries in the family”) and their consecutive presentation in the questionnaire. Reflecting this particular methodological issue—by allowing the items to correlate beyond the factorial structure—emended the misfit. A subsequent CFA on the slightly reversed measurement model performed in both groups supported the assumed categories indicated by an acceptable fit (59) in the German [χ2_(108)_ = 222.70, CFI = 0.924, RMSEA = 0.065, SRMR = 0.059] as well as in the Turkish group [χ2_(108)_ = 163.53, CFI = 0.922, RMSEA = 0.058, SRMR = 0.059].

Table 2 provides an overview of the specific causal attributions composing the mentioned categories. We created factor-based scores for each category and reversed them in order for high values to indicate high agreement with the respective attributional category.

**Table 2:** Attribution categories and the composing items with corresponding standardized factor loadings for both subsamples.

**Items**	**1**	**2**	**3**	**4**	**5**
**NATIVE GERMANS**					
**Factor 1: Biogenetic causes**					
Disease of the brain	0.648				
Heredity	0.470				
Drug abuse	0.643				
**Factor 2: Childhood adversities**					
Grew up in a broken home		0.638			
Unkind treatment at the parental home		0.733			
Sexual abuse		0.694			
Shock caused by a dramatic life event		0.559			
**Factor 3: Current stress**					
Strains and worries in the partnership			0.673		
Strains and worries in the family			0.741		
Work related strains and worries			0.523		
Unconscious conflict			0.827		
Poverty			0.748		
Pressure to fulfill the expectations of others			0.580		
**Factor 4: Supernatural causes**					
Possessed by demons				0.929	
A punishment by God				0.699	
**Factor 5: Unfavorable traits**					
Immoral lifestyle					0.797
weakness of will					0.570
**TURKISH IMMIGRANTS**					
**Factor 1: Biogenetic causes**					
Disease of the brain	0.610				
Heredity	0.453				
Drug abuse	0.656				
**Factor 2: Childhood adversities**					
Grew up in a broken home		0.511			
Unkind treatment at the parental home		0.685			
Sexual abuse		0.688			
Shock caused by a dramatic life event		0.469			
**Factor 3: Current stress**					
Strains and worries in the partnership			0.710		
Strains and worries in the family			0.726		
Work related strains and worries			0.439		
Unconscious conflict			0.719		
Poverty			0.652		
Pressure to fulfill the expectations of others			0.590		
**Factor 4: Supernatural causes**					
Possessed by demons				0.829	
A punishment by God				0.716	
**Factor 5: Unfavorable traits**					
Immoral lifestyle					0.948
Weakness of will					0.505

#### Emotional Responses

Participants were furthermore requested to indicate their agreement to a list of 10 statements of possible emotions evoked by the person presented in the vignette. Statements such as “the person frightens me” or “the person makes me feel uneasy” were rated on a 5-point Likert scale ranging from 1 = “completely agree” to 5 = “completely disagree.” Previous studies using the same or a similar approach found a three-factored structure of emotional reactions, both by exploratory principal-component analyses (8, 60) and confirmatory factor analyses (61). The factors identified were “fear,” “anger,” and “prosocial,” the latter being also referred to as “pity” in previous publications. CFAs performed on the two groups separately yielded the same three factors with an adequate fit in both groups [χ2_(32)_ = 77.45, CFI = 0.934, RMSEA = 0.072, SRMR = 0.068 in the German group and χ2_(32)_ = 51.86, CFI = 0.934, RMSEA = 0.069, SRMR = 0.064 in the Turkish immigrant group]. We consecutively created factor scores for further analyses and reversed them in order for high numerical values to indicate high emotional reactions.

#### Desire for Social Distance

In order to assess the extent of social rejection of people with mental health issues, we implemented the *Social Distance Scale* [SDS, (17)]. Participants were requested to indicate how willingly they would accept the person described in the vignette across 7 social situations on a 5-point Likert scale ranging from complete agreement to complete disagreement. The social situations included renting the described person a room, accepting her as a colleague, as a neighbor, letting her take care of one's children, having her marry into one's family, introducing her to friends and recommending her for a job. The SDS is commonly used in population surveys on social rejection (10) and shows high internal consistencies between.75 and 0.90 (39). In line with those findings, Cronbach's alpha in our German and Turkish immigrant subsamples was.87 and.88, respectively. A CFA performed to verify a unifactored structure resulted in an initial misfit. A subsequent analysis revealed the misfit in both groups to originate from an item pair with very similar degree of social distance (accepting the person as a neighbor and accepting her as a colleague) as opposed to the other social situations queried which are socially closer and rather located in the private sphere (e.g., introducing the person to friends). Allowing the items to correlate beyond the factorial structure corrected the misfit. A subsequent CFA on the slightly reversed measurement model performed in both groups yielded an acceptable fit^2^ in both the German [χ2_(13)_ = 50.57, CFI = 0.949, RMSEA = 0.098, SRMR = 0.039] and the Turkish group [χ2_(13)_ = 40.27, CFI = 0.943, RMSEA = 0.110, SRMR = 0.045]. Here too, we calculated a scale score. High numerical values indicate a high desire for social distance.

### Statistical Analyses

All statistical analyses were carried out using R version 3.2.5. CFAs were computed using the latent variable analysis (lavaan) package version 0.5-23.1097 of R (62). Regarding our sample size of *n* < 500, according to Weston and Gore (63) the following indices and cutoff values were considered an acceptable overall fit: Comparative Fit Index (CFI >0.90), Root-Mean-Square Error of Approximation (RMSEA < 0.10), and Standardized Root Mean Residual (SRMR < 0.10).

Paired *t*-tests were carried out to test for differences within groups between means for both vignettes combined. Independent *t*-tests were performed to examine differences between vignettes within both groups. Since the criterion of variance homogeneity is not met for most of the applied comparisons, we used Welch's modification which does not assume equal variance. *P*-values were adjusted for multiple comparisons using the Benjamini-Hochberg method as it allows for higher preservation of power than the conservative Bonferroni correction (64). Effect sizes Cohen's *d* were calculated in order to delineate the magnitude of inspected differences. To facilitate a better understanding of the relationships between variables Pearson's zero-order correlations are displayed when deemed helpful.

To examine the relationship between the desire for social distance and causal beliefs as well as the mediation by emotional reactions, path analysis models were performed in both subsamples separately for each vignette condition. We determined direct effects, total indirect effects and total effects by computing the corresponding products and sums of products (65, 66). Coefficients were adjusted for the effect of gender, age and education.

### Missing Data

The dataset contained 1.69% of missing values. Although not being a significant fraction, due to their even distribution a case wise deletion of all cases containing missing values would lead to the loss of about 47% of all cases. This would not only greatly impair statistical power of the subsequent analyses but also potentially bias the results in an unpredictable way if the missing values are not completely at random [MCAR; (67)]. The MCAR-assumption is very strong and hardly ever applies in practice. Therefore, the missing values were imputed. We used the *mice* package version 2.46.0 in R following the instructions of the package's author (68). Mice automatically specifies a regression-based imputation model for each of the variables depending on data type by using all relevant predictors available in the data set. It furthermore adds prediction error into the regression yielding more plausibly distributed imputed values than deterministic regression would do through the consideration of noise (69, 70).

The imputed data sets were later pooled to create a single complete data set, rendering our approach technically a single imputation. Although generally, multiple imputations are preferred over a single imputation, single imputation methods have been shown to perform equally well if the overall number of missing values is small, as in the case our data set (71). All presented analyses are performed on the imputed data set.

#### Measurement Invariance

The invariance of applied measures is a prerequisite for meaningful cross-cultural comparisons in order to ensure that equivalent constructs have been measured across the particular groups (72). Therefore, prior to analyzing cultural differences in our sample, we assessed configural, metric, and scalar invariance of the applied scales. Configural invariance is given when factors, as well as the pattern of relationships between factors and their indicators, are identical across both groups. We established configural invariance by performing CFAs for each construct of interest in both samples separately (see scale descriptions above) as well as in both groups combined.

Metric invariance is given when factor loadings are identical across both groups. Only with equal factor loadings can the constructs be assumed to have the same meaning across administrations. Metric invariance is required in order to interpret differences in relationships of variables, such as correlations and regression weights, across cultures (73). Scalar invariance is indicated by equal intercepts of indicators across groups and is required in order to compare differences in factor mean levels across groups and interpret them as meaningful differences. Metric and scalar invariance were assessed by adding constraints of equivalence to the measurement model in accordance with Cheung and Rensvold (74). We subsequently examined the changes of fit indices, expecting the change due to addition of constraints not to exceed the following dimensions ΔCFI = 0.010, ΔRMSEA = 0.010 (74, 75).

## Results

### Measurement Invariance

In order to ensure that group differences can be interpreted as meaningful differences across the two subsamples, measurement invariance was tested for each of the scales implemented. A summary of the results is presented in Table 3. Configural invariance was established for all three constructs. When loadings were restricted to be equal in both groups model fit did not worsen for causal beliefs and the SDS, but showed significant worsening for emotional reactions [Δ^*x*2^_(7)_ = 14.83, *p* = 0.038]. However, since the differences of fit indices CFI and RMSEA lay within the dimensions recommended by Cheung and Rensvold (74), metric invariance can be assumed for all three constructs. Given the equality of loadings, differences in the relationships of variables such as correlations and regression weights in our sample can be understood to represent meaningful cross-cultural differences. Subsequently, we restricted the item intercepts to be equal across both subsamples. This implementation resulted in a substantial misfit amongst all three constructs, thereby demonstrating a lack of scalar invariance for the applied measures. Hence, differences in the means of variables across both subsamples might result from divergent item difficulty and not represent meaningful cross-cultural differences. We therefore abstain from comparisons of means across both subsamples.

**Table 3:** Summary of test for measurement invariance.

**Construct**	**Invariance**	***^**x2**^(df)***	**CFI**	**RMSEA**	**Δ^x2^**	***p*-value**	**ΔCFI**	**ΔRMSEA**
Causal beliefs	Configural	451.87 (216)	0.918	0.058				
	Metric	461.22 (228)	0.924	0.054	5.61	0.934	+0.006	−0.004
	Scalar	516.36 (240)	0.906	0.059	54.97	< 0.001^***^	−0.018	+0.005
Emotional reaction	Configural	157.20 (64)	0.932	0.063				
	Metric	176.36 (71)	0.923	0.064	14.84	0.039^*^	−0.009	+0.001
	Scalar	257.37 (78)	0.845	0.086	161.31	< 0.001^***^	−0.078	+0.022
Desire for social distance	Configural	113.70 (30)	0.940	0.098				
	Metric	122.30 (34)	0.936	0.095	7.98	0.083	−0.004	−0.003
	Scalar	250.84 (40)	0.840	0.138	122.31	< 0.001^***^	−0.096	+0.043

* *p < 0.05*,

*** *p < 0.001*.

### Descriptive Results and Differences Within Subsamples

#### Causal Attributions

Table 4 provides an overview of the agreement to the different categories of causal attribution in each group for both vignettes combined as well as separately for depression and schizophrenia. In both subsamples, causal attributions to current stress and childhood adversities are the most prevalent for both vignettes combined. Supernatural causes, on the other hand, received the least endorsement in both groups. While biogenetic causes and childhood adversities are equally frequently endorsed in the German sample [*t*_(301)_ = 1.35, *p* = 0.179], in the Turkish immigrant subsample biogenetic causes are less frequently assumed to be causal for mental illness than childhood adversities [*t*_(172)_ = −7.32, *p* < 0.001]. At the same time Turkish immigrants rate unfavorable traits to be as likely a cause of mental illness as biogenetic causes [*t*_(172)_ = 1.84, *p* = 0.076]. In contrast, agreement to unfavorable traits in the German subsample is far lower than to biogenetic causes [*t*_(301)_ = −17.77, *p* < 0.001].

**Table 4:** Summary of causal attributions and comparison of causal attributions between depression and schizophrenia vignettes within both groups.

		**Depression**	**Schizophrenia**	***t*-statistics**	**Adjusted *p*-values**	***D***
	***M* (*SD*), *n* = 302**	***M* (*SD*), *n* = 145**	***M* (*SD*), *n* = 157**			
**NATIVE GERMANS**						
Biogenetic causes	3.34 (0.77)	3.10 (0.78)	3.57 (0.69)	*t*_(289.50)_ = 5.61	< 0.001^***^	0.64
Childhood adversities	3.42 (0.79)	3.49 (0.79)	3.35 (0.79)	*t*_(289.06)_ = 1.60	0.138	−0.18
Current stress	3.50 (0.68)	3.80 (0.56)	3.21 (0.66)	*t*_(297.61)_ = 8.45	< 0.001^***^	−0.97
Supernatural causes	1.42 (0.77)	1.39 (0.70)	1.45 (0.83)	*t*_(297.54)_ = 0.75	0.454	0.09
Unfavorable traits	2.12 (0.93)	2.21 (0.96)	2.03 (0.90)	*t*_(293.93)_ = 1.70	0.138	−0.20
		**Depression**	**Schizophrenia**	***t*****-statistics**	**Adjusted** ***p*****-values**	***D***
	***M*** **(*****SD*****)**, ***n*** **=** **173**	***M*** **(*****SD*****)**, ***n*** **=** **87**	***M*** **(*****SD*****)**, ***n*** **=** **86**			
**TURKISH IMMIGRANTS**						
Biogenetic causes	3.34 (0.89)	3.19 (0.91)	3.49 (0.84)	*t*_(170.31)_ = 2.26	0.063	0.34
Childhood adversities	3.80 (0.76)	3.77 (0.76)	3.83 (0.76)	*t*_(170.92)_ = 0.55	0.586	−0.08
Current stress	3.81 (0.67)	3.91 (0.62)	3.71 (0.72)	*t*_(166.41)_ = 1.98	0.081	−0.30
Supernatural causes	2.13 (1.16)	1.85 (0.97)	2.42 (1.26)	*t*_(159.18)_ = 3.32	0.006^**^	0.50
Unfavorable traits	3.18 (0.97)	3.13 (0.90)	3.23 (1.04	*t*_(166.85)_ = 0.72	0.586	0.11

** *p < 0.01*,

*** *p < 0.001*.

As for the comparison across vignettes, in the German subsample, biogenetic explanations were more frequently endorsed in the schizophrenia condition compared to the depression condition (*d* = 0.64). Current stress, in turn, was rather endorsed to cause depression than for schizophrenia (*d* = −0.97). These findings are in line with results reported by previous research. Interestingly, no equivalent differences could be observed in the Turkish immigrant subsample. However, Turkish immigrants showed higher agreement to supernatural causes for schizophrenia than to depression (*d* = 0.50), while the agreement in the German subsample remained equally low across both vignettes.

Table 5 illustrates zero-order correlations between the categories of causal attributions in both groups. As can be seen, in the Turkish immigrant subsample all categories were correlated which was not the case for the German subsample. All observed correlations in both groups were positive.

**Table 5:** Correlations between the categories of causal beliefs in both subsamples.

	**Native Germans**					**Turkish immigrants**				
	**1.**	**2.**	**3.**	**4.**	**5.**	**1.**	**2.**	**3.**	**4.**	**5.**
1. Biogenetic causes	1.00					1.00				
2. Childhood adversities	0.26	1.00				0.51	1.00			
3. Current stress	–	0.55	1.00			0.29	0.50	1.00		
4. Supernatural causes	–	–	–	1.00		0.29	0.31	0.16	1.00	
5. Unfavorable traits	–	0.26	0.21	0.32	1.00	0.22	0.18	0.28	0.38	1.00

*Only significant correlations are displayed (p < 0.05)*.

#### Emotional Reactions

People in both subsamples experienced more prosocial emotions than fear [*t*_(301)_ = 19.77, *p* < 0.001 and *t*_(172)_ = 9.56, *p* < 0.001 in the German and Turkish immigrant subsample, respectively] and more fear than anger [*t*_(301)_ = 16.22, *p* < 0.001 and *t*_(172)_ = 12.69, *p* < 0.001 in the German and Turkish immigrant subsample, respectively]. Thus, in both groups a prosocial emotional reaction was the most prevalent, which is consistent with previous findings.

In the German subsample, all emotional reactions differed in their intensity across the depression and schizophrenia vignettes. Germans experienced more fear (*d* = 0.76) and anger (*d* = 0.36) and less prosocial emotions (*d* = −0.52) when confronted with the description of a schizophrenic person compared to a person suffering from depressive symptoms. Interestingly, while Turkish immigrants similarly experienced more fear (*d* = 0.73) and anger (*d* = 0.38) in response to the schizophrenia vignette, their prosocial emotions did not differ between vignettes. See Table 6 for an overview of means and comparisons between vignettes.

**Table 6:** Summary of emotional reactions and comparison between depression and schizophrenia in both groups.

		**Depression**	**Schizophrenia**	***t*-statistics**	**Adjusted *p*-values**	***D***
	***M* (*SD*), *n* = 302**	***M* (*SD*), *n*= 145**	***M* (*SD*), *n*= 157**			
**NATIVE GERMANS**						
Fear	2.44 (0.97)	2.07 (0.89)	2.77 (0.93)	*t*_(299.48)_ = 6.64	< 0.001^***^	0.76
Anger	1.63 (0.64)	1.51 (0.57)	1.74 (0.68)	*t*_(297.55)_ = 3.10	0.002^**^	0.36
Prosocial	3.86 (0.68)	4.04 (0.60)	3.70 (0.71)	*t*_(297.74)_ = −4.54	< 0.001^***^	−0.52
		**Depression**	**Schizophrenia**	***t*****-statistics**	**Adjusted** ***p*****-values**	***D***
	***M*** **(*****SD*****)**, ***n*** **=** **173**	***M*** **(*****SD*****)**, ***n*** **=** **87**	***M*** **(*****SD*****)**, ***n*** **=** **86**			
**TURKISH IMMIGRANTS**						
Fear	2.74 (1.16)	2.35 (0.97)	3.14 (1.20)	*t*_(162.87)_ = 4.80	< 0.001^***^	0.73
Anger	1.78 (0.76)	1.64 (0.69)	1.93 (0.80)	*t*_(166.55)_ = 2.51	0.013^*^	0.38
Prosocial	3.79 (0.68)	3.80 (0.67)	3.78 (0.68)	*t*_(166.41)_ = −1.98	0.869	−0.02

* *p < 0.05*,

** *p < 0.01*,

*** *p < 0.001*.

#### Desire for Social Distance

Desire for social distance was higher for schizophrenia than for depression in the German [*M* = 3.52, *SD* = 0.77 vs. *M* = 2.95, *SD* = 0.74, *t*_(299.55)_ = 6.57, *p* < 0.001, *d* = 0.76] and in the Turkish immigrant subsample [*M* = 3.49, *SD* = 0.90 vs. *M* = 3.21, *SD* = 0.88, *t*_(170.75)_ = 2.07, *p* = 0.040, *d* = 0.32] alike.

### Path Analysis

**Figure 1:**
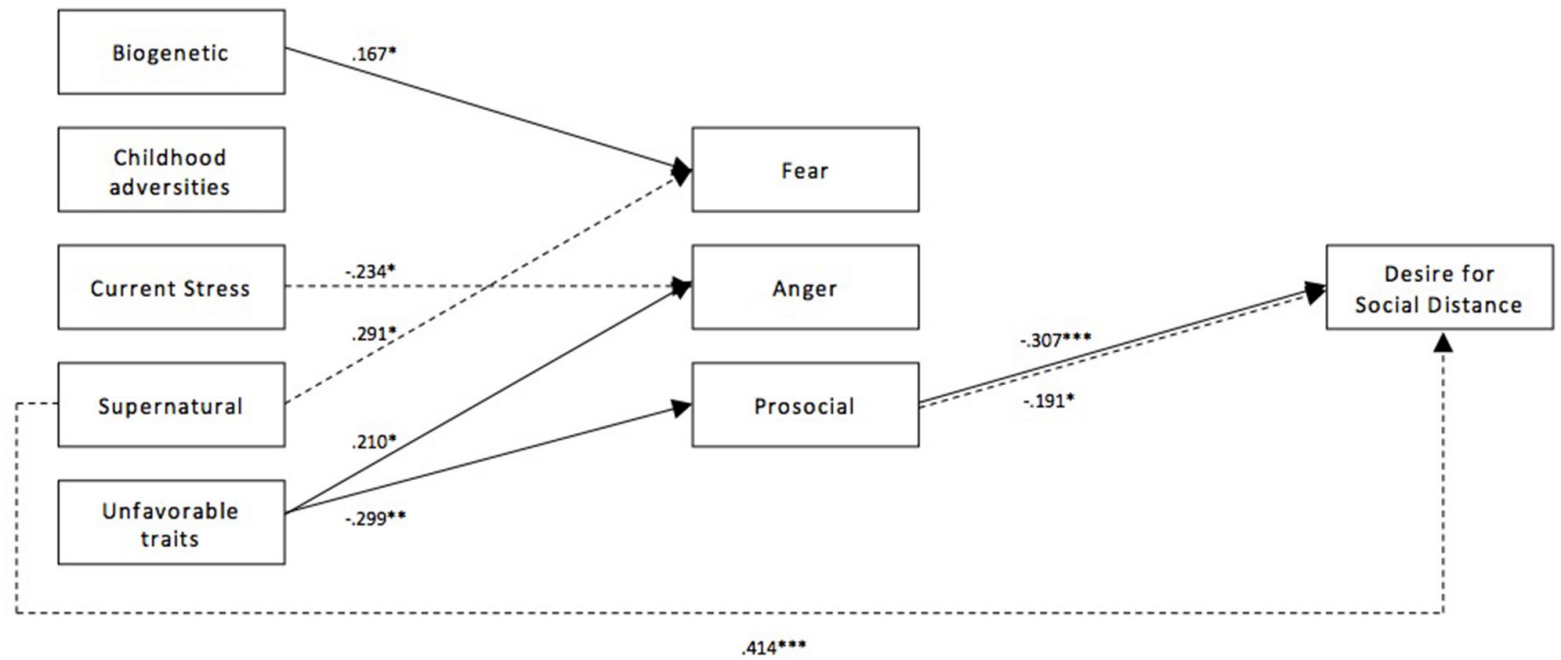
Path model of the relationship between causal beliefs, emotional reactions, and desire for social distance for depression for both subsamples. Paths of the German subsample (*n* = 302) are depicted with a solid line, paths for the Turkish immigrant subsample (*n* = 173) are depicted with a dashed line. Presented path coefficients are standardized path coefficients β; only significant coefficients are reported. ^*^p < 0.05, ^**^p < 0.01, ^***^p < 0.001.

**Figure 2:**
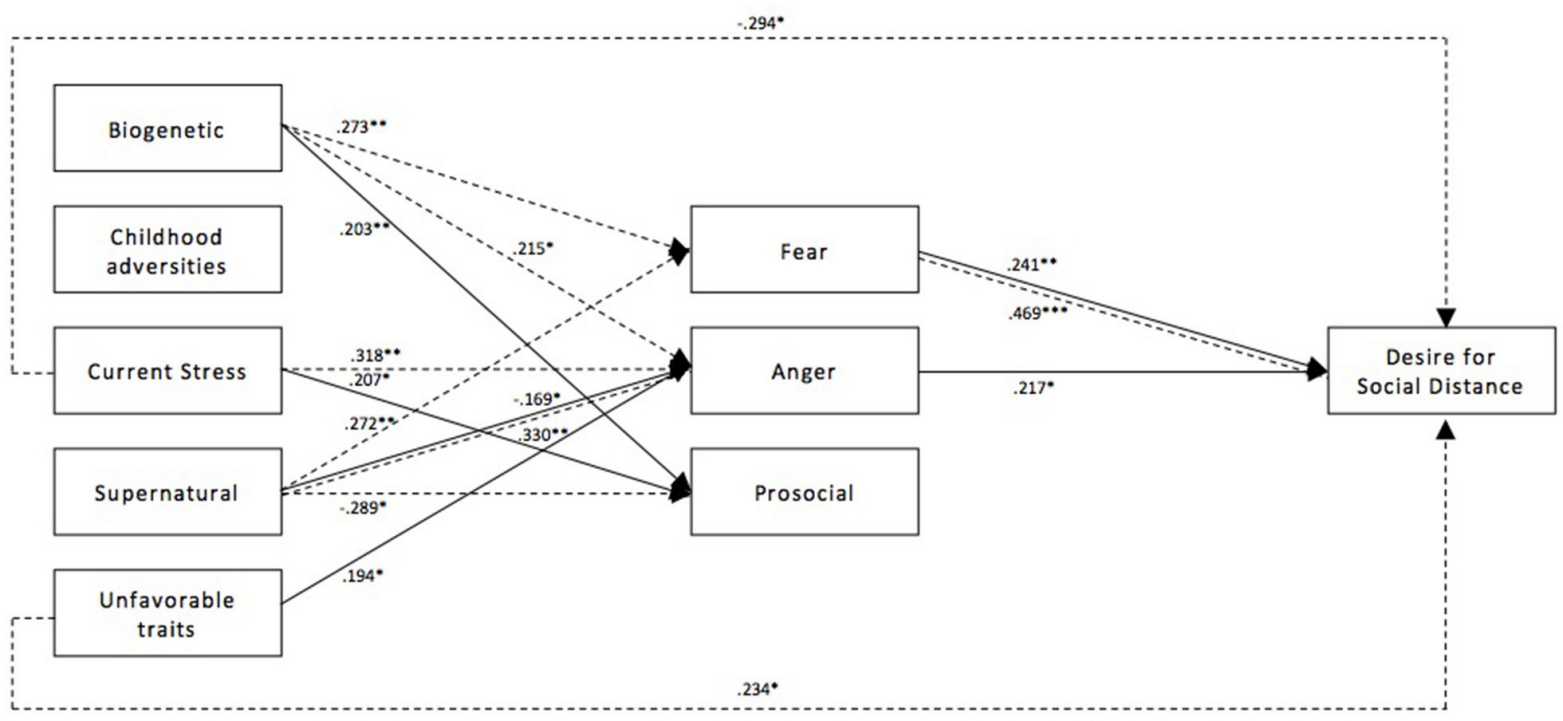
Path model of the relationship between causal beliefs, emotional reactions, and desire for social distance for schizophrenia for both subsamples. Paths of the German subsample (*n* = 302) are depicted with a solid line, paths for the Turkish immigrant subsample (*n* = 173) are depicted with a dashed line. Presented path coefficients are standardized path coefficients β; only significant coefficients are reported. ^*^p < 0.05, ^**^p < 0.01, ^***^p < 0.001.

Figures 1, 2 illustrate the path model between causal beliefs, emotional reactions and desire for social distance for depression and schizophrenia, respectively. Only significant paths are depicted along with their corresponding standardized path coefficients (β*)*. In the depression vignette, the tested model explained 13.9% of the variance of desire for social distance in the German subsample and 33.6% in the Turkish subsample. In the schizophrenia vignette, the proportion of explained variance of desire for social distance was 25.8% in the German and 23.0% in the Turkish subsample.

#### Depression Vignette

In the German subsample, biogenetic causal beliefs were associated with higher fear (β = 0.167) while no such connection could be observed in the Turkish immigrant subsample. Attribution of the depicted condition to childhood adversities, current stress or supernatural causes had no significant connection with any of the emotional reactions in the German subsample. However, in the Turkish immigrant subsample current stress was associated with reduced anger (β = −0.234) and supernatural causal beliefs were associated with increased fear (β = 0.291). Moreover, supernatural causal beliefs were also directly related to an increased desire for social distance in the Turkish subsample (β = 0.414) whereas the same path failed to reach significance in the German subsample (β = 0.128, *p* = 0.061). Unfavorable traits were related to stronger anger (β = 0.210) and less prosocial emotions (β = −0.299) in the German subsample, while no connection could be seen in the Turkish subsample. Surprisingly, a positive association between fear and desire for social distance was established in neither of the groups. However, in the Turkish immigrant group, a trend toward a positive connection was recognizable (β = 0.201, *p* = 0.051). This was not the case for the German subsample (β = 0.093, *p* = 0.327). Prosocial emotions led to less desire for social distance in both subsamples (β = −0.307 and β = −0.191 for the German and Turkish immigrant subsample, respectively), while anger did not affect the desire for social distance in neither of the two groups.

Unfavorable traits were the only causal attribution to have a significant total indirect effect on desire for social distance in the German subsample (β = 0.092, *p* = 0.031), but its total effect was not significant (β = 0.118, *p* = 0.065). In the Turkish subsample, no causal attribution had a significant total indirect effect on desire for social distance, yet there was a significant positive total effect of supernatural causal beliefs (β = 0.486, *p* < 0.001). See Table 7 for an overview of significant direct, total indirect and total effects of causal belief on the desire for social distance.

**Table 7:** Overview of direct effects, total indirect effects, and total effects of causal beliefs on desire for social distance for both vignettes across both subsamples.

	**Native germans**	**Turkish immigrants**
**DIRECT EFFECTS**		
Depression	–	Supernatural ▴
Schizophrenia	–	Current stress ▾ Intrapersonal ▴
**TOTAL INDIRECT EFFECTS**		
Depression	Intrapersonal ▴	–
Schizophrenia	Intrapersonal ▴	Biogenetic ▴ Supernatural ▴
**TOTAL EFFECTS**		
Depression	–	Supernatural ▴
Schizophrenia	Intrapersonal ▴	Current stress ▾

*Only significant effects are listed*.

#### Schizophrenia Vignette

Biogenetic causal beliefs were associated with increased fear (β = 0.273, *p* = 0.007) in the Turkish immigrant subsample, but, while a similar trend did appear, the path missed significance in the German subsample (β = 0.174, *p* = 0.076). Similarly, the endorsement of biogenetic causal beliefs was related to more anger in the Turkish subsample (β = 0.226, *p* = 0.033). However, while a similar trend was observed in the German subsample, it failed to reach significance (β = 0.149, *p* = 0.066). Biogenetic causal beliefs were linked to more prosocial emotions in the German (β = 0.203, *p* = 0.005), but not in the Turkish immigrant subsample (β = −0.068, *p* = 0.469). As in the depression vignette, childhood adversities were not connected with emotional reactions or desire for social distance in either of the two subsamples. Attribution of schizophrenia to current stress was associated with increased anger in the Turkish immigrant subsample (β = 0.318, *p* = 0.006). At the same time attribution to current stress had a negative direct effect on desire for social distance (β = −0.294, *p* = 0.005). While no such effects were observed in the German subsample, attribution to current stress was instead associated with more prosocial emotions (β = 0.207, *p* = 0.011) here. Interestingly, supernatural causal beliefs were found to be related to less anger in the German subsample (β = −0.169, *p* = 0.031), while conversely a relation with more anger could be observed in the Turkish immigrant subsample (β = 0.330, *p* = 0.004). In line with these supernatural causal beliefs were associated with less prosocial emotions among Turkish immigrants (β = −0.289, *p* = 0.027). Unfavorable traits were connected to more anger in the German subsample (β = 0.252, *p* = 0.019). In the Turkish subsample, unfavorable traits were not connected to emotional reactions, but instead were directly associated with an increased desire for social distance (β = 0.234, *p* = 0.025).

Among Germans, no total indirect, as well as no total effect on desire for social distance, was found for biogenetic causal beliefs, childhood adversities, current stress and supernatural causal beliefs. Attribution of schizophrenia to unfavorable traits was the only causal belief to have a significant total indirect effect (β = 0.082, *p* = 0.031) as well as a total effect (β = 0.225, *p* = 0.004), being associated with higher social distance. In the Turkish immigrant subsample, we found a positive total indirect effect of biogenetic causal beliefs on the desire for social distance (β = 0.161, *p* = 0.022) which was mainly driven by the increase of fear and its increase of stigma (β = 0.469, *p* < 0.001). However, the total effect of biogenetic beliefs did not reach significance. Similarly, supernatural causal beliefs had a significant total indirect effect on the desire for social distance in the Turkish subsample (β = 0.135, *p* = 0.001), but no total effect could be shown (β = 0.069, *p* = 0.574). Conversely, current stress did not have a total indirect effect, but showed a reducing total effect on desire for social distance (β = −0.335, *p* = 0.003).

The control variables gender and education had no effect on the constructs of interest neither for subsamples nor for vignettes. However, in the German subsample, higher age was associated with less prosocial emotions and a higher desire for social distance in both vignette conditions and with a higher perception of anger in the schizophrenia condition. In the Turkish immigrant subsample age was associated with more fear in both vignette conditions and with an increased the desire for social distance toward a person with schizophrenia.

## Discussion

Our study aimed at investigating the influence of causal beliefs on stigma of mental illness mediated by emotional reactions. Further, we intended to draw cross-cultural comparisons between Germans and Turkish immigrants in Germany, thereby extending the knowledge on this matter in immigrant samples which are still underrepresented in stigma research. In summary, we found associations between causal beliefs of mental illness, emotional reactions to it and people's desire to distance themselves from an affected person in both subsamples. The extent of stigmatization of the mentally ill is influenced by the attribution of causes of mental illness. This influence is mediated by the emotional reactions evoked. Furthermore, the present study found cross-cultural differences in the pattern of causal beliefs as well as their relationship to individual stigma.

In the German subsample, biogenetic causal attributions were as frequently endorsed as psychological causes (e.g., current stress and childhood adversities), Turkish immigrants expressed more agreement to the latter. They furthermore considered unfavorable traits to be as likely a cause of mental illness as biogenetic causes. These findings about Turkish immigrants largely correspond to a study by Utz (54) on a Turkish sample in Turkey, where mental illness was most frequently attributed to similar psychological causes, and biogenetic causal beliefs were approximately as prevalent as unfavorable traits. In contrast, Germans endorsed unfavorable traits far less than biogenetic factors, which is in accordance with the findings of Angermeyer on Germans (14). Since only metric measurement invariance could be established, no mean comparisons between cultural groups were drawn. However, the mentioned relative difference in endorsement indicates more importance of biogenetic explanations among Germans. Causal beliefs appeared to be more illness specific in the German than in the Turkish subsample. Turkish immigrants rather attributed depression and schizophrenia to similar causes while German participants held more differentiated causal explanations depending on the disorder in question. It points in the same direction that in the Turkish subsample all causal belief categories were positively interrelated, which was not the case for German participants. These differences point to a more narrowly defined explanatory model of mental illness of German respondents.

In both subsamples, participants reported more fear and anger when confronted with the schizophrenia vignette compared to the depression vignette, which is in accordance with previous findings (55). Interestingly, a differential pattern could be observed regarding prosocial emotions. In the German subsample, participants indicated more prosocial emotions toward a person suffering from depression compared to schizophrenia, but in the Turkish immigrant subsample, prosocial emotions did not differ between vignettes. Prosocial emotions toward a mentally ill person, like pity and the desire to help, were not related to specific categories of mental illness attributions among Turkish immigrants as opposed to German natives.

As predicted, desire for social distance was higher for schizophrenia than for depression in both groups, a finding that has been reported quite consistently in the past (10). Regarding the relationship between causal beliefs and stigma, the single causal belief to have a meaningful total effect on stigma in the German subsample was the attribution of schizophrenia to unfavorable traits, which was related to higher desire for social distance. Moreover, in the Turkish immigrant sample only supernatural causal beliefs had a significant total effect on desire for social distance. These were associated with an increased desire for social distance, and attribution to current stress, which in turn was associated with less desired distance. All three effects are in accordance with the hypothesized direction. However, contrary to our hypotheses both effects found in the Turkish sample did not emerge in the German subsample. Moreover, supernatural causal beliefs may also have failed to be significantly related to stigma in the German sample due to their very low endorsement in the first place.

Surprisingly, the hypothesized total effect of biogenetic beliefs on stigma appeared in neither of the cultural groups, which contrasts findings in other samples (23, 29). Overall, a potential link between biogenetic causal beliefs and emotions showed a somewhat inconclusive pattern. For Germans confronted with the depression vignette, biogenetic beliefs were associated with more fear but not the other emotional reactions, namely anger and prosocial emotions. While this suggests a negative impact of biogenetic causal beliefs for depression, an indirect effect of biogenetic beliefs on stigma did not emerge. Interestingly, for the schizophrenia vignette biogenetic causal beliefs were connected to neither fear nor anger, but instead to increased prosocial emotions. This indicates a positive impact of biogenetic causal beliefs for schizophrenia, but again no significant indirect effect on stigma could be found. This is explained mainly with prosocial emotions not being linked to the desire for social distance. In other words, the increase of prosocial emotions did not translate into reduced stigma in this case. In the Turkish subsample in the depression vignette biogenetic causal beliefs were not related to any emotion, while in schizophrenia they increased fear and anger as we predicted for the German subsample based on previous studies (23, 29). Thus, in the Turkish subsample, biogenetic causal beliefs were associated with endorsement of fear and anger which in turn increased stigma.

Remarkably, causal beliefs showed a direct effect on desire for social distance in the Turkish immigrant subsample. In the depression condition, supernatural causal beliefs directly led to a greater desire for social distance, whereas in the schizophrenia condition unfavorable traits were associated with higher and current stress was associated with less desire for social distance. Emotional reactions did not mediate the effects of these causal beliefs. No direct, unmediated effects were observed in the German subsample. Corrigan's concept of stigma implies that prejudices, which are comprised of the cognitive and affective agreement to negative stereotypes, are preceding stigmatizing behavior (15). While we found some evidence in favor of this concept in both subsamples, it appears to be less appropriate to describe the occurrence of stigma in the Turkish immigrant subsample. This is indicated by the above mentioned direct effects of causal beliefs which are not mediated by emotional reactions in the Turkish immigrant subsample.

Following this, it was an unexpected finding that in both subsamples emotional reactions were not always related to the desire for social distance. In their work, Schomerus et al. (29) found evidence for other mediators of the influence of causal beliefs on stigma, such as the responsibility for falling ill and recovering, notions of dangerousness, differentness and expected treatability. The proportion of explained variance of desire for social distance of this model was comparable to the findings in our study. Future studies could look at more comprehensive models encompassing a broader set of mediator variables to get to a more detailed understanding of the mechanisms involved in the development of stigma.

In total, a few inconsistencies of our results remain, particularly regarding the effect of biogenetic causal beliefs and, in some cases, the missing mediational value of emotional reactions. Moreover, the observed correlations are in many cases of explorative nature. Replications and expansion are therefore required to provide further clarification of the relationship between causal beliefs and stigma and its mediation by emotional reaction as well as to ensure the validity of our results. This is of particular importance in case of our Turkish immigrant subsample. With the attempt to recruit participants of a more difficult to reach migrant sample for the first time, our study has to be understood as a first step toward an understanding of causal beliefs and associated attitudes and stigma in Turkish and also other immigrants in Germany.

However, in a qualitative study on causal beliefs, Vardar finds the variation within the examined Turkish immigrant group to be as high as the variation between Turkish and German participants (51). Immigrants of the same origins could be experiencing very different realities of everyday life and be subjected to disparate standards, norms and subcultures, resulting in a high heterogeneity in attitudes toward a wide range of issues, including mental illness. Moreover, different levels of acculturation and assimilation, language skills as well as self-perceived cultural belonging further contribute to the outlined heterogeneity. Thus, a generalization of observed differences would not do this complexity justice. Furthermore, the mentioned difference in the realities of everyday life might be at least partly responsible for the cross-cultural differences we found between Germans and Turkish immigrants. Considering other interdependent factors named above might provide further insight.

From previous research, we were aware of lower participation rates of ethnic minorities in health research studies as potential participation barriers are commonly reported (57). Therefore, aiming for an urban sample recruited in Berlin, we had to consider sampling methods which take into account the difficulties in accessing Turkish migrants. At the same time, we intended to maintain a balance between representativity of the subsamples on the one hand and comparability of the samples on the other. As Dingoyan et al. (57) reported potential reasons for the lower participation rates of Turkish immigrants in Germany (e.g., lack of knowledge, lack of interest and trust, anxiety regarding data privacy protection) we applied the recruitment strategies recommended by the these authors to enhance participation rates. These strategies were namely word-of-mouth promotion, contacting Turkish key figures such as religious leaders, teachers or doctors as well as tangible incentives and trust building through transparent communication of the project and its conditions. Further, we decided to gather data on the Turkish sample first and recruit a comparable German sample in a second step. This objective could be achieved in terms of sociodemographic characteristics such as age, gender, civil status, employment rates, and proportion of university students, where samples did not differ significantly. Further, some regional characteristics of the population of Berlin in comparison to the general population of Germany are represented in the data of included participants. For instance, in Berlin the proportion of singles is higher than in the general population in Germany, which is represented in both samples equally (76, 77). Also, regardless of their ethnic background, on average persons living in Berlin have a lower level of income and significantly less religious affiliations than the general German population (76, 77). It should be noted that the level of education in both samples was high and deviated significantly from the general population of native Germans as well as Turkish immigrants. As this was the case in both subsamples, this factor probably had no confounding effect between groups, but the possible influence of a high educational level must be considered as a limitation in the interpretation of the study results.

In total, with this sampling strategy, we managed to get access to a group of people, namely Turkish immigrants, which is difficult to access, by maintaining relative comparability with the urban population of Berlin. Further research would be needed to replicate the cross-cultural differences emerged in the present study and to further investigate why these differences occur.

## Limitations

Our study comes with some limitations. Firstly, though the applied path model assumes a certain direction of effect, it is important to point out that our study design does not allow for testing causality, since it involves only one measurement point and no corresponding experimental manipulation. However, while there is little experimental research on how a change in causal beliefs affects public attitude, the evidence that does exist supports the hypothesized direction. For example, Walker and Read (78) indicate that the promotion of psychological causes in contrast to biogenetic causes leads to less perception of dangerousness of mentally ill persons. Secondly, the proportion of variance in the desire for social distance explained by our model is rather low, ranging between 13.9 and 33.6% for depression and between 25.8 and 23.0% for schizophrenia in the German and Turkish subsamples, respectively. Apparently, as mentioned above, other factors may play a role in the explanation of the desire for social distance, such as the mediators investigated by Schomerus et al. (29), but also familiarity with mental illness (79) and labeling the symptoms described in the vignette as a mental illness (61). However, the explanatory power of the present work is comparable with that found in previous studies.

A further concern is the low response rate, especially among Turkish immigrants, which entails the risk of limited representability of the assessed sample and the above mentioned high education status of participants present in both subsamples.

A direct informal addressing of possible participants in public spaces, as well as later indirect acquisition through intermediary units, resulted in the impossibility to register response rates and characteristics of the non-respondents. Evidently, due to the challenges that result from the limited access to Turkish immigrants open for academic research, as discussed earlier, minor deviations from ideal representability were to be expected. Furthermore, the inclusion of a Turkish immigrant subsample provides an important and long-awaited contribution to the broadening of knowledge on causal beliefs and stigma in ethnic minorities in Western countries, especially under consideration of the vast underrepresentation of ethnic minorities in this research field. The reason for treating first- and second generation Turkish participants as one group is the assumption that both subgroups were under the influence of another than the German culture in their upbringing. What is also common in the first and the second generation of Turkish immigrants in Germany is the experience of living as a cultural minority in a host country and being treated as a migrant (as opposed to a native German). We hypothesize that in the Turkish sample these experiences may have led to different norms and value systems than native Germans which in turn may have resulted in the differences described in this study when it comes to causal beliefs and stigma. To investigate these issues in more detail future studies could investigate different subsamples, e.g., separating immigrants from Turkey and Germans born and raised in Germany of Turkish origin.

Our work is meant to serve as a sound basis for further research in this area. Future research could apply similar path models on Turkish citizens in Turkey and on comparisons between different ethnicities, different generations of migrants and migration regimes. By these means, possible differences between Turkish and Turkish immigrants in Western countries as well as between first- and second generation immigrants in Germany could be explored investigating the specific role of immigration and belonging to a minority in the country of residence.

Nonetheless, our study provides valuable insights into the mechanisms of culture-dependent pathways between causal beliefs, emotions and stigmatizing behavioral intent. By utilizing rigorous statistical methods, we aimed to derive robust results and strengthen confidence in our findings. Firstly, we examined all scales with confirmatory factor analyses, establishing the validity of both the applied measures and the conclusions drawn. Secondly, with thoroughly assessing measurement invariance of the implemented scales, we did provide further evidence for the cross-cultural applicability of the scales administered. Thirdly, the examination by means of a path analysis allowed for a better understanding of mediating mechanisms behind the relation between causal beliefs and stigma. In addition, as has been mentioned above, the present study is of importance due to its consideration of an immigrant subsample.

## Conclusion

The results of the present study enabled us to draw implications for future anti-stigma interventions. From what we found, it became apparent that the endorsement of supernatural beliefs and unfavorable traits is particularly problematic since it was related to higher intentions for stigmatizing behavior. Moreover, although biogenetic causal beliefs could not be shown to be positively associated with desire for social distance, they were also not associated with less stigmatization, as has been theorized for a long time (25, 80). Biogenetic explanations were however linked to more fear and anger in some cases, which is in accordance with the results of more recently conducted studies (16, 29, 33). Educational programs that challenge the aforementioned causal attributions consequently seem to be an appropriate intervention and might be effective in reducing individual discriminating behavior toward people suffering from mental illness. Furthermore, we found multiple differences in the effects of causal beliefs on stigma between cultural groups and vignettes. These differences suggest that differential target group and illness specific anti-stigma interventions might prove more useful. For instance, our results indicate that the emphasis of current stress as a cause might prove helpful in confronting stigma in schizophrenia. Attribution to current stress had no impact on stigma in depression, a finding also reported by Schomerus et al. (29). While promoting current stress as a cause for schizophrenia seems promising to reduce stigma, promoting current stress as a cause for depression might have no effect. Similarly, anti-stigma campaigns that deliberately target beliefs as unfavorable traits in Germans or supernatural causal beliefs in Turkish immigrants could prove to be more effective interventions than more generic forms. Since causal attributions did not explain a very high proportion of variance of stigma, it might also be useful to look for appropriate interventions outside of changing causal beliefs. For instance, studies show that interventions that focus on familiarity and direct contact between patients with mental illness and persons not affected can increase social acceptance and reduce stigmatizing attitudes (81, 82).

Stigma poses an additional burden for people suffering from mental illness, one that often impairs their social participation and can prevent them from seeking adequate help. It is therefore important to understand how stigma develops to counteract it by setting up evidence-based anti-stigma interventions. Our study found evidence for cross-cultural differences in causal attribution patterns and the relationships between causal beliefs and stigma, as well as between depression and schizophrenia. Differential anti-stigma interventions could pose a considerable approach.

## Author Contributions

UV: study design, data collection, data evaluation, writing, and editing the manuscript. JG: study design, data collection, data evaluation, and editing the manuscript. DW, AW, and SH: data collection, data evaluation, and editing the manuscript. MB, GS, and MA: study design and editing the manuscript. EH: study design, data evaluation, and editing the manuscript.

### Conflict of Interest Statement

The authors declare that the research was conducted in the absence of any commercial or financial relationships that could be construed as a potential conflict of interest.

## Footnotes

^1^In the present study the term “immigrant” refers to individuals, who migrated from Turkey themselves (first generation) as well as their descendants (second generation) (50).

^2^Please note, that the RMSEA is exceeding the threshold of.10 in the Turkish group. However, since both other indicators indicate a fairly good fit, we decided for acceptance of the measurement model.
